# Supplementary material for: A human antibody selective for transthyretin amyloid removes cardiac amyloid through phagocytic immune cells
Source: Nat Commun. 2021 May 25;12:3142. doi: 10.1038/s41467-021-23274-x (PMC8149704; doi:10.1038/s41467-021-23274-x)
Supplement: Supplementary file 1 — Supplementary Information [file 41467_2021_23274_MOESM1_ESM.pdf]

Supplementary information

Title: **A human antibody selective for transthyretin amyloid removes cardiac amyloid through phagocytic immune cells**

Aubin Michalon*[1], Andreas Hagenbuch[1], Christian Huy[1], Evita Varela[1], Benoit Combaluzier[1], Thibaud Damy[2], Ole B. Suhr[3], Maria J. Saraiva[4], Christoph Hock[1,5], Roger M. Nitsch[1,5], Jan Grimm[1]

Supplementary Fig. 1

Epitope mapping using overlapping peptide array. The sequences of the peptides bound by NI301A are indicated, with the common sequence identifying the binding epitope shown in bold. Two point mutations present in the peptides 38 and 40 are indicated in green color.

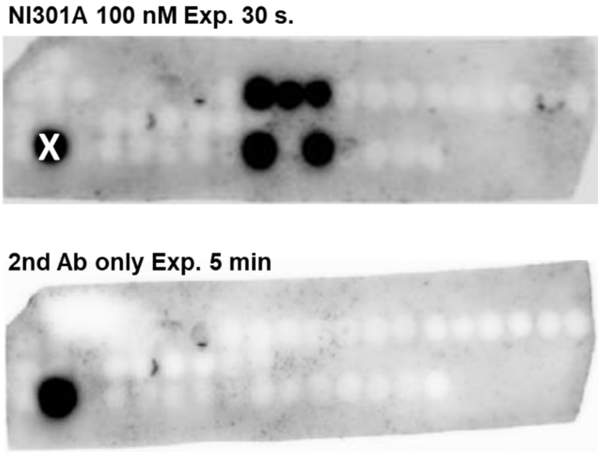

09 FRKAADDT**WEPFA**SG
10 ADDT**WEPFA**SGKTSE
11 **WEPFA**SGKTSESGEL
38 INVAVHVFRNAADDT**WEPFA**
40 AADDT**WEPF**NSGKTSESGEL

Supplementary Fig. 2

TTR immunoprecipitation experiment (Fig.1H): images of the entire western blot membranes.

TTR selective bands run at 14 kDa for TTR monomers and ~30 kDa for TTR dimers. In addition, human heavy chains (~50 kDa) are detected by the protein A-HRP detection system. There are two sources of human heavy chains in this experiment, the NI301A and isotype control antibodies used for IP (but not Dako A0002, which is a rabbit polyclonal antibody), and the human IgGs present in human serum which are carried over in a serum concentration dependent manner.

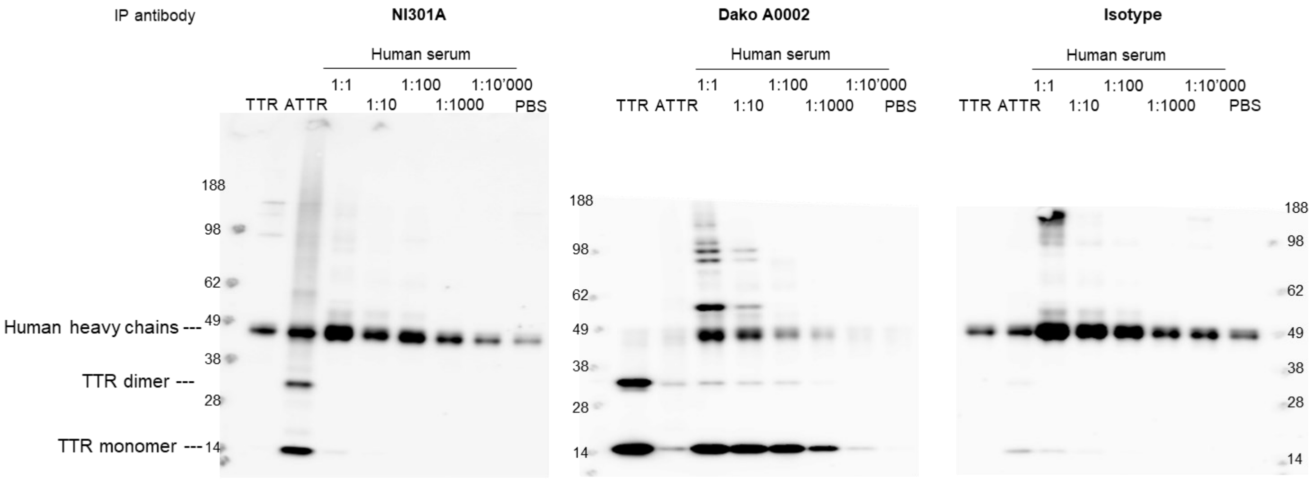

Supplementary Fig. 3

Long exposure duration of the western blot presented in Figure 3 did not reveal any unspecific binding of NI301A to the non-TTR proteins presents in the amyloid fibril extract.

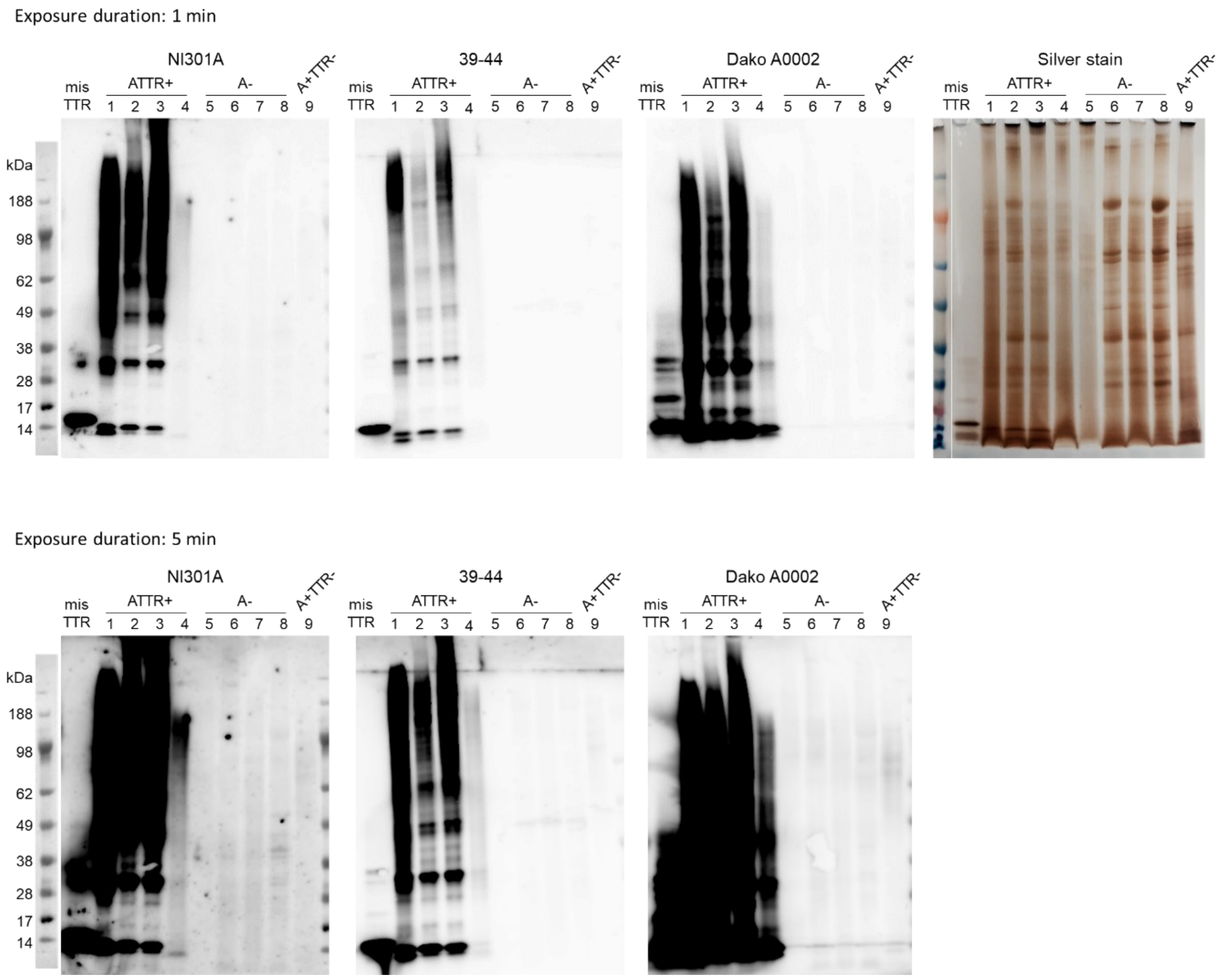

Supplementary Fig. 4

Evaluation of NI301A binding selectivity by immunohistochemistry on human fresh frozen tissue arrays. The arrays contained 90 tissue sections representing 30 different organs each from three different individuals. An ATTR+ myocardium tissue section was added on each slide as positive control. Representative images for ATTR+ heart (positive control) and liver, heart and nerve normal tissues.

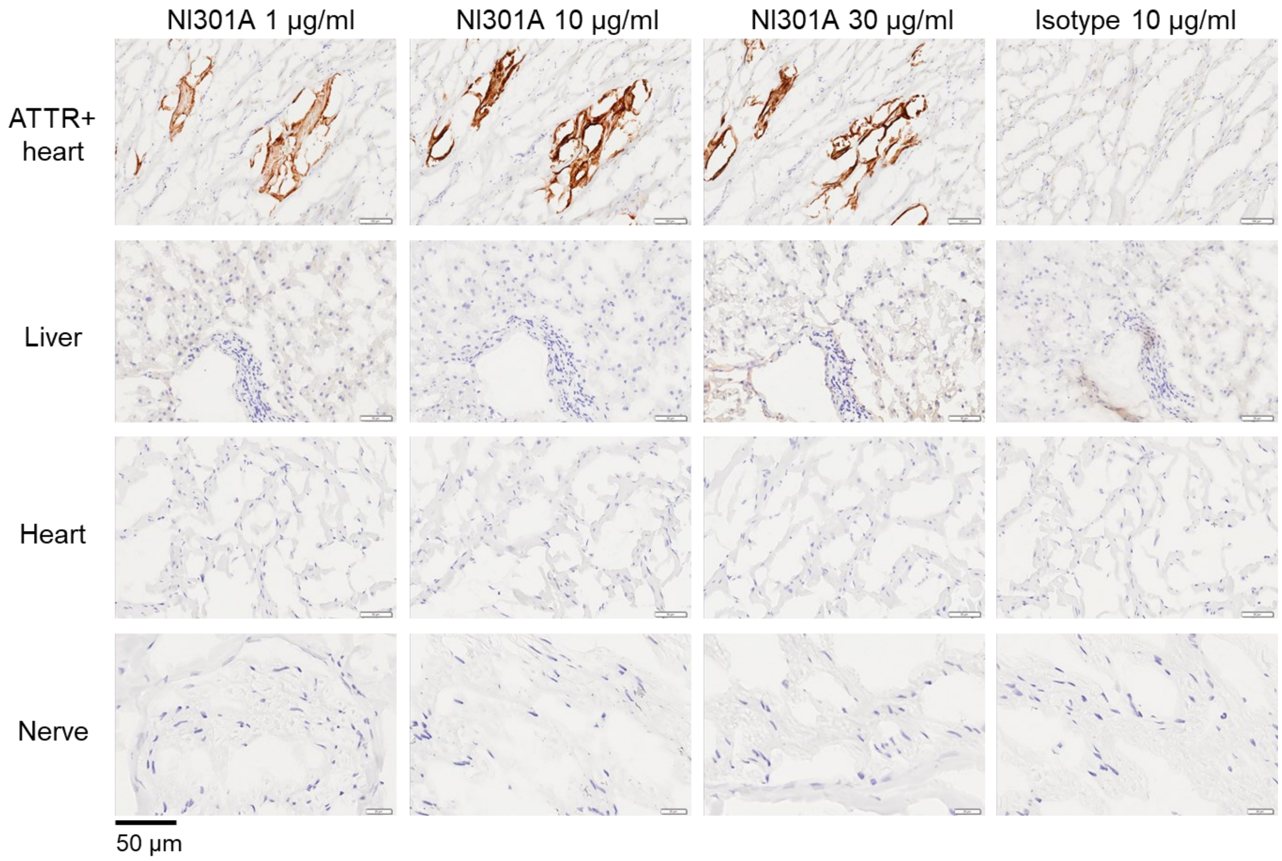

Supplementary Fig. 5

Gating strategy to determine the percentage of macrophages double positive for fluorescently conjugated NI301A antibody (NI301A-550) and fluorescently conjugated ATTR-L55P aggregates (ATTR-L55P-488). Plots are presented for the following conditions: 1) ATTR-L55P 7 µg/mL, no antibody; 2) ATTR-L55P 7 µg/mL and NI301A 10 nM; 3) ATTR-L55P 7 µg/mL and NI301A 80 nM; 4) NI301A 80 nM, no ATTR.

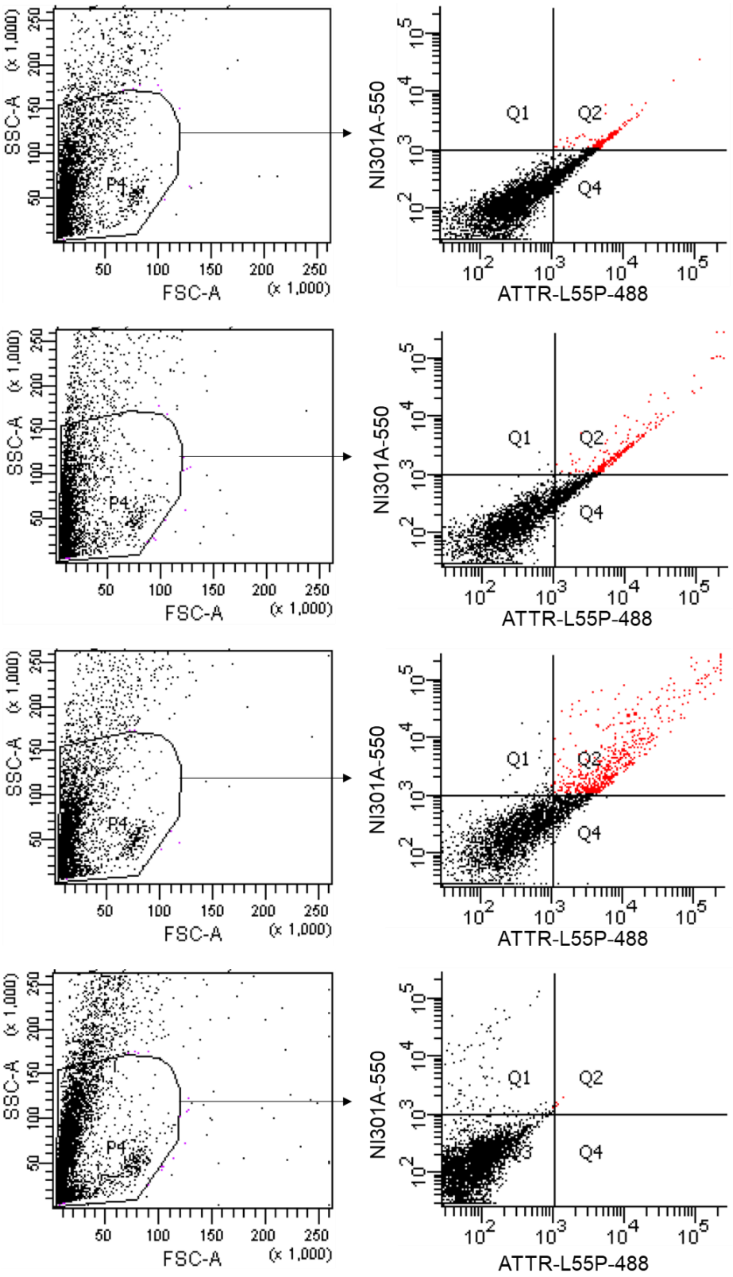

Supplementary Fig. 6

Characterization of cells infiltrating the ATTR fibril grafts for the expression of CD11B, LY6G and IBA1.

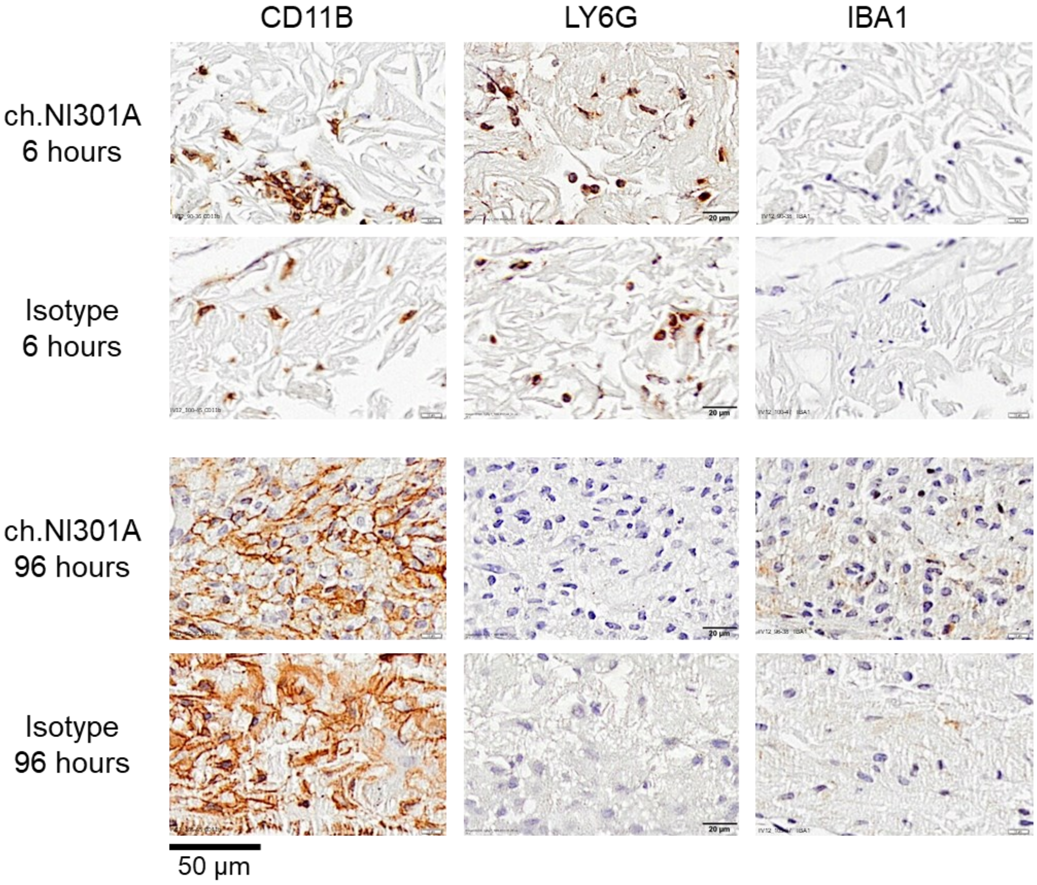

Supplementary Fig. 7

Exemplary staining of autopsy samples from patients with heart failure analyzed for presence of ATTR amyloidosis using the amyloid dye congo red and immunohistochemistry with NI301A biotin conjugate.

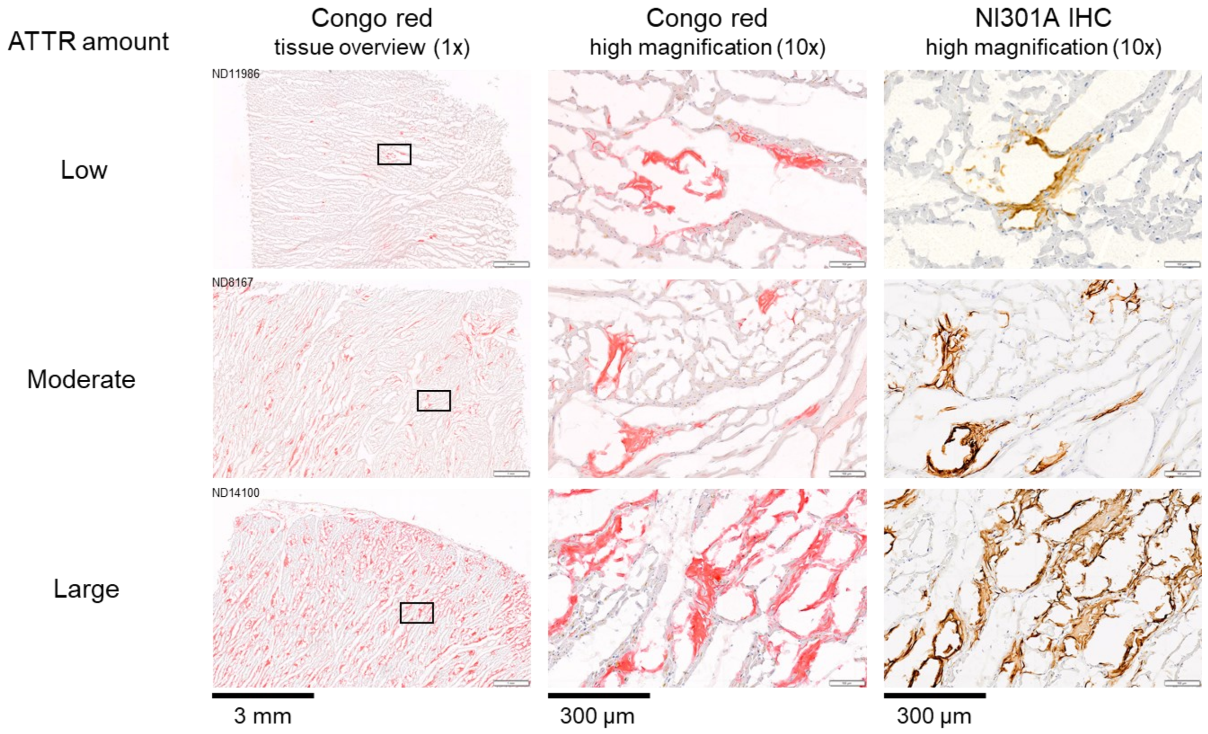

Supplementary Table 1

List of autopsy samples from patients with heart failure analyzed for presence of ATTR amyloidosis. All subjects are males over 80 years old.

N°	ID	DISEASE	CAUSE OF DEATH	ORIGIN	ATTR	ATTR AMOUNT
1	ND08018	Norany	Subdural hematoma	C	-	0
2	**ND08167**	Norany	Delirium	C	+	large
3	ND08226	Norany	Respiratory failure	C	-	0
4	ND08560	Cardio	Cardiopulmonary arrest	C	-	0
5	**ND08587**	Norany	Heart failure	C	+	large
6	ND08820	Norany	Cardiac arrest	C	-	0
7	ND08848	Norany	Cardiac arrest	C	-	0
8	**ND08970**	Norany	Congestive heart failure	C	+	moderate
9	ND09164	Norany	Heart failure	C	-	0
10	ND09310	Norany	Respiratory arrest	C	-	0
11	ND08819	Norany	Cardiac arrest	C	-	0
12	ND09378	Norany	Cardiac arrest	C	-	0
13	ND09430	Cardio	Cardiac related	C	-	0
14	**ND09796**	Cardio	Cardiopulmonary arrest	C	+	low
15	ND09802	Cardio	Respiratory arrest	C	-	0
16	ND09809	Cardio	Prostate cancer	C	-	0
17	ND09835	Cardio	Cardiopulmonary arrest	C	-	0
18	ND09951	Cardio	Cardiac arrest	C	-	0
19	ND09965	Cardio	Cardiac arrest	B	-	0
20	**ND10001**	Cardio	Respiratory failure	C	+	moderate
21	**ND10150**	Cardio	Respiratory arrest	C	+	large
22	**ND10183**	Cardio	Cardiac arrest	C	+	large
23	**ND10234**	Cardio	Cardiac arrest	C	+	large
24	ND10338	Cardio	Cardiac arrest	C	-	0
25	**ND10351**	Cardio	Cardiac arrest	C	+	moderate
26	ND10386	Cardio	Respiratory failure	C	-	0
27	ND10501	Cardio	Cardiac arrest	C	-	0
28	ND10507	Cardio	Cardiopulmonary arrest	C	-	0
29	ND10703	Cardio	Cardiac arrest	C	+	large
30	**ND10800**	Cardio	Cardiac arrest	C	+	moderate
31	ND10871	Cardio	Congestive heart failure	C	-	0
32	ND10878	Cardio	Congestive heart failure	C	-	0
33	ND10884	Cardio	Cardiac arrest	C	+	low
34	ND11132	Cardio	Pneumonia	C	+	low
35	ND11182	Cardio	Disease process	C	+	low
36	ND11313	Cardio	Cardiovascular arrest	C	-	0
37	ND11370	Cardio	Cardiac arrest	B	-	0
38	ND11386	Cardio	Cardiac	C	-	0
39	ND11753	Cardio	COPD	C	-	0
40	ND11759	Cardio	Cardiovascular arrest	C	-	0
41	ND11882	Cardio	Ruptured aneurysm	C	-	0
42	**ND11986**	Cardio	Cardiovascular arrest	C	+	low
43	ND11989	Cardio	Respiratory arrest	C	-	0
44	ND12029	Cardio	Cardiac arrest	C	-	0

45	ND12091	Cardio	Multisystem organ failure	C	-	0
46	ND12102	Cardio	Pneumonia	C	-	0
47	**ND12192**	Cardio	Respiratory failure	C	**+**	large
48	ND12326	Cardio	Cardiopulmonary arrest	C	-	0
49	ND12327	Cardio	Cardiac arrest	C	-	0
50	ND12346	Cardio	Congestive heart failure	C	-	0
51	ND12351	Cardio	Stroke	C	-	0
52	ND12372	Cardio	Encephalopathy	C	-	0
53	**ND12389**	Cardio	Cardiac arrest	C	**+**	low
54	ND12403	Cardio	Respiratory failure	C	-	0
55	ND12609	Cardio	Respiratory failure	C	-	0
56	ND12661				**+**	low
57	ND12679	Cardio	Unspecified	C	**+**	low
58	ND12709	Cardio	Pneumonia	C	**+**	low
59	ND12492	Cardio	Unspecified	C	**+**	low
60	ND12690	Cardio	Intraventricular hemorrage	C	-	0
61	**ND13067**	Cardio	Cardiovascular arrest	C	**+**	large
62	ND13134	Cardio	Head trauma	C	-	0
63	ND13238	Cardio	Heart failure	C	**+**	low
64	ND13388	Cardio	Heart failure	C	-	0
65	ND13510	Cardio	Respiratory failure	C	-	0
66	ND13567	Cardio	Intracerebral hemorrhage	C	-	0
67	ND13646	Cardio	Cardiac arrest	C	-	0
68	ND13734	Cardio	Cardiac arrest	C	-	0
69	ND13745	Cardio	Myocardial infarction	C	-	0
70	ND13754	Cardio	Atherosclerosis	C	-	0
71	ND13769	Cardio	Respiratory arrest	C	-	0
72	ND13887	Cardio	Cardiac/Respiratory failure	C	-	0
73	ND13918	Cardio	Cardiac arrest	C	-	0
74	ND13980	Cardio	Respiratory failure	C	-	0
75	ND14027	Cardio	Congestive heart failure	C	-	0
76	ND14034	Cardio	Cardiac arrest	C	-	0
77	**ND14100**	Cardio	Stroke	B	**+**	large
78	ND14131	Cardio	Congestive heart failure	C	-	0
79	ND14183	Cardio	Cardiogenic shock	C	-	0
80	ND14276	Cardio	Cardiac arrest	C	-	0
