# Supplementary material for: A human antibody selective for transthyretin amyloid removes cardiac amyloid through phagocytic immune cells
Source: Nat Commun. 2021 May 25;12:3142. doi: 10.1038/s41467-021-23274-x (PMC8149704; doi:10.1038/s41467-021-23274-x)
Supplement: Supplementary file 3 — Reporting Summary [file 41467_2021_23274_MOESM3_ESM.pdf]

nature research

Corresponding author(s): Aubin Michalon

Last updated by author(s): Feb 24, 2021

Reporting Summary

Nature Research wishes to improve the reproducibility of the work that we publish. This form provides structure for consistency and transparency in reporting. For further information on Nature Research policies, see our Editorial Policies and the Editorial Policy Checklist.

Statistics

For all statistical analyses, confirm that the following items are present in the figure legend, table legend, main text, or Methods section.

n/a	Confirmed	
☐	☒	The exact sample size (n) for each experimental group/condition, given as a discrete number and unit of measurement
☐	☒	A statement on whether measurements were taken from distinct samples or whether the same sample was measured repeatedly
☐	☒	The statistical test(s) used AND whether they are one- or two-sided<br>*Only common tests should be described solely by name; describe more complex techniques in the Methods section.*
☐	☒	A description of all covariates tested
☐	☒	A description of any assumptions or corrections, such as tests of normality and adjustment for multiple comparisons
☐	☒	A full description of the statistical parameters including central tendency (e.g. means) or other basic estimates (e.g. regression coefficient) AND variation (e.g. standard deviation) or associated estimates of uncertainty (e.g. confidence intervals)
☐	☒	For null hypothesis testing, the test statistic (e.g. F, t, r) with confidence intervals, effect sizes, degrees of freedom and P value noted<br>*Give P values as exact values whenever suitable.*
☒	☐	For Bayesian analysis, information on the choice of priors and Markov chain Monte Carlo settings
☒	☐	For hierarchical and complex designs, identification of the appropriate level for tests and full reporting of outcomes
☒	☐	Estimates of effect sizes (e.g. Cohen's d, Pearson's r), indicating how they were calculated

Our web collection on statistics for biologists contains articles on many of the points above.

Software and code

Policy information about availability of computer code

Data collection	Plate reader: ThermoFisher SkanIt 6.02<br>Binding kinetics: ForteBio DataAcquisition 8.2<br>Flow cytometry: BD FACSAria II<br>Microscopy: Olympus OlyVIA 2.9
Data analysis	Binding kinetics: ProteOn Manager 2.0 and ForteBio DataAnalysis 8.2<br>Flow cytometry: FACS Diva version 6.1.3<br>Microscopy: MediaCybernetics Image Pro Premier 9.3.<br>Data management: Microsoft Excel (version 2101)<br>Statistical analysis: GraphPad Prism 7 and 8

For manuscripts utilizing custom algorithms or software that are central to the research but not yet described in published literature, software must be made available to editors and reviewers. We strongly encourage code deposition in a community repository (e.g. GitHub). See the Nature Research guidelines for submitting code & software for further information.

Data

Policy information about availability of data

All manuscripts must include a data availability statement. This statement should provide the following information, where applicable:

- Accession codes, unique identifiers, or web links for publicly available datasets
- A list of figures that have associated raw data
- A description of any restrictions on data availability

Source data are provided with this paper as a supplementary excel file.

Field-specific reporting

Please select the one below that is the best fit for your research. If you are not sure, read the appropriate sections before making your selection.

☒ Life sciences ☐ Behavioural & social sciences ☐ Ecological, evolutionary & environmental sciences

For a reference copy of the document with all sections, see nature.com/documents/nr-reporting-summary-flat.pdf

Life sciences study design

All studies must disclose on these points even when the disclosure is negative.

Sample size	Sample size was determined based on prior knowledge of intrinsic and extrinsic variability and practical considerations (Button K, 2013; Krzywinski D, 2013). No calculation of sample size and statistical power was performed in absence of prior knowledge about the expected effect size.<br>For ex vivo tissue amyloid phagocytosis assays, results obtained during method development indicated that 6 sections per condition was appropriate to average out the section to section variability and remain practical with all conditions investigated in parallel in the same experiment. Similarly, the in vivo efficacy studies were conducted using the largest group size group compatible with running all treatment groups in parallel in the same study.
Data exclusions	No data point was excluded from the analyses, but in some cases samples were not quantifiable, e.g. tissue loss during histological processing.
Replication	All main effects reported in this study were observed repeatedly using identical or similar study designs. NI301A activity on tissue amyloid removal was observed >=5 times using different patient tissues and PBMCs from different donors. ch.NI301A activity in vivo was also observed in >=5 studies. Both in vivo and ex vivo dose-response studies were performed at least twice using similar but not identical antibody concentrations.
Randomization	For all studies, sample processing and quantification steps were randomized to ensure absence of technical artifacts.
Blinding	The ex vivo experiments were conducted with the experimentalist blinded to the treatment applied. Blinding was also maintained during quantification of amyloid load in tissues, which was performed semi-automatically using an image analysis software, and during data QC.<br>The in vivo studies were conducted using blinding to group allocation during tissue collection, processing, staining, quantification and QC.

Reporting for specific materials, systems and methods

We require information from authors about some types of materials, experimental systems and methods used in many studies. Here, indicate whether each material, system or method listed is relevant to your study. If you are not sure if a list item applies to your research, read the appropriate section before selecting a response.

Materials & experimental systems

n/a	Involved in the study	
☐	☒	Antibodies
☒	☐	Eukaryotic cell lines
☒	☐	Palaeontology and archaeology
☐	☒	Animals and other organisms
☐	☒	Human research participants
☒	☐	Clinical data
☒	☐	Dual use research of concern

Methods

n/a	Involved in the study	
☒	☐	ChIP-seq
☐	☒	Flow cytometry
☒	☐	MRI-based neuroimaging

Antibodies

Antibodies used	Primary antibodies:<br>NI301A, ch.NI301A and ch.NI301A-LALAPG, Neurimmune AG (proprietary reagents, various lots used)<br>Isotype antibodies (43A11 and ch.43A11), Neurimmune (proprietary reagents, various lots used)

	Anti-Transthyretin 39-44, Alexotech, AT-550-01<br>Anti-human prealbumin antibody, Dako, A0002<br>CD11B, Abcam ab133357<br>LY6G, Abcam ab2557 (clone NIMP-R14)<br>IBA1, Wako 019-19741<br><br>Secondary antibodies:<br>Anti-human IgG Fcγ (unlabelled), Jackson Immunoresearch, 709-005-098<br>Anti-human IgG HRP-conjugated, Jackson Immunoresearch, 709-036-098<br>Anti-rabbit IgG HRP-conjugated, Jackson Immunoresearch, 111-035-045<br>Anti-mouse IgG HRP-conjugated, Jackson Immunoresearch, 115-035-146
Validation	NI301A and variants: Validated for ELISA, WB and IHC (cf. manuscript, especially Fig.1, 2 and 3)<br>Isotype antibodies: Validated for ELISA, WB, IF and IHC (Jelcic, 2015)<br>Anti-Transthyretin 39-44: Validated for ELISA, WB and IHC. (Goldsteins, 1999; Palha, 2001; Sousa, 2002)<br>Anti-human prealbumin antibody, Dako, A0002: Validated for ELISA, WB, IP and IHC.<br>CD11B, Abcam ab133357: Validated for WB, IF and IHC<br>LY6G, Abcam ab2557 (clone NIMP-R14): Validated for IHC.<br>IBA1, Wako 019-19741: Validated for ICC and IHC

Animals and other organisms

Policy information about studies involving animals; ARRIVE guidelines recommended for reporting animal research

Laboratory animals	SKH1 Elite female mice 2.5 - 3 month old.
Wild animals	The study did not involve wild animals.
Field-collected samples	The study did not involve samples collected from the field.
Ethics oversight	Animal studies were approved by the cantonal veterinary office (license ZH269-14).

Note that full information on the approval of the study protocol must also be provided in the manuscript.

Human research participants

Policy information about studies involving human research participants

Population characteristics	De-identified blood samples for antibody screening were obtained from healthy elderly donors.<br>Autopsy heart tissues were obtained from 80+ years old males with heart failure with preserved ejection fraction.
Recruitment	Healthy elderly blood donors were recruited by the University of Zurich.<br>Autopsy heart tissues were collected by the National Disease Research Interchange.
Ethics oversight	Human blood samples were collected under informed consent and approval by the Ethics Committee of the Canton of Zurich.<br>Human heart tissues were collected under informed consent and use approved by the NDRI.

Note that full information on the approval of the study protocol must also be provided in the manuscript.

Flow Cytometry

Plots

Confirm that:

- [x] The axis labels state the marker and fluorochrome used (e.g. CD4-FITC).
- [x] The axis scales are clearly visible. Include numbers along axes only for bottom left plot of group (a 'group' is an analysis of identical markers).
- [ ] All plots are contour plots with outliers or pseudocolor plots.
- [x] A numerical value for number of cells or percentage (with statistics) is provided.

Methodology

Sample preparation	Macrophages were differentiated from fresh human monocytes by culturing these for 10-15 days in macrophage serum-free medium (M-SFM, Life technologies) supplemented with macrophage colony-stimulating factor (100 ng/mL M-CSF, Miltenyi). Phagocytosis was done for 2 hours at 37°C in the presence of fucoidan, ATTR-L55P labeled with Atto488 (Sigma), NI301A labeled with Atto550 (Sigma) at concentrations from 0 to 80 nM.
Instrument	BD FACSAria II

Software	FACS Diva version 6.1.3
Cell population abundance	The number of double positive cells in the Q2 quadrant was determined and used for quantification.
Gating strategy	Cells were gated according to their size on the SSC-A/FSC-A graph and then plotted for FITC (ATTR-L55P-Atto488) and PE (NI301A-Atto550) to determine the number of double positive cells.

☒ Tick this box to confirm that a figure exemplifying the gating strategy is provided in the Supplementary Information.
